# Modeling of changes in the nuclide composition of VVER reactor fuel using artificial neural network

**DOI:** 10.1016/j.heliyon.2024.e26228

**Published:** 2024-02-10

**Authors:** Prince Asabi Boakye, Alexey Goryunov Germanovich

**Affiliations:** a Tomsk Polytechnic University Research Institute of Nuclear Physics, Tomsk, Russia; b Head, Department of Nuclear Fuel Cycle, Tomsk Polytechnic University, Oblast, Russia

**Keywords:** ANFIS, Uranium fuel, Neural network, Nuclides concentration, MATLAB

## Abstract

The paper seeks to give a computer-based model, using an artificially intelligent technique. This is the adaptive neuro-fuzzy inference system (ANFIS), to predict the concentration changes of certain nuclides in the uranium fuel used for VVER reactors. It uses low-enriched uranium dioxide as a fuel in its solid state. The reactivity in the core is controlled by the control rods. Nuclide concentration changes in the reactor fuel, if not monitored, may cause the unsafe operation of the reactor. Hence, the need for this study. The nuclides considered in this study are, U-235, U-236, U-237, U-238, Pu-239, Pu-240, Pu-241, Am-242 and Am-243. The initial computational technique was performed using MATLAB Simulink. The simulation data for all the concentrations of the nuclides were obtained. Then the proposed ANFIS model was performed and tested using data from the Simulink. Results from the simulink and ANFIS were compared and the results were in good agreement. Again, the results were compared to the Calculating Actinide Inventory (CAIN) code from the IAEA-TECHDOC-1535 published in 2007 and both showed a good agreement. An RMSE of about 0.98% and 1.25% were obtained for training and testing data respectively. The developed model will allow technologists to quickly perform calculations for the reactor, which is essential for safety systems. It could be concluded that the ANFIS model can effectively be used to predict the concentration of each nuclide in the uranium fuel because it is effective, precise with lesser error, and does not consume time.

## List of abbreviations

VVER: Water water energy reactor
PWR: Pressurized water reactor
BWR: Boiling water reactor
RCS: Reactor coolant system
CPS: Control and protective system
SG: Steam generator
FA: Fuel assembly
RPV: Reactor pressure vessel
RV: Reactor vessel
EFPD: Effective full power days
CR: Conversion ratio
NPP: Nuclear power plant
AIS: Artificial intelligent system
NN: Neural network
NCN: Neural computing network
FL: Fuzzy logic
GA: Genetic algorithm
ANFIS: Adaptive network fuzzy inference system
P_MD_: Moderator effect
CZP: Cold zero power
HZP: Hot zero power
RMSE: Root mean square error
K: multiplication factor

## 1 Introduction

The generation IV reactor designs have posed many reasons why better developments of systems of operation and engineering should be enacted [1]. Energy produced in a Nuclear Power Plant (NPP) is by thermal fission. The neutron flux changes with time and space. This causes changes in the concentrations of nuclides in the uranium fuel. Excessive concentrations of both fissile and fertile nuclides realized in the uranium fuel may result in unsafe rector operation and subsequently cause NPP accidents. This study seeks to give a prediction of the change in concentration of the major nuclides emitted and their right proportion needed for safe reactor operation as the uranium fuel undergoes fission [2]. We shall consider a program code in MATLAB software. Designing our specific work in Simulink blocks, generating the code from there, and later taking the data for training [3]. The equation of flux, equation of change in the concentration of fissile material, and equation of flux transient due to a positive step reactivity insertion were used. The code will be generated from the simulation and this code will be compared to another international research [4]. The advantage of this code is, it is possible to be simulated in the shortest time and still will give a precise solution because of the complex nature of the numerical methods used. The study of reactor Kinetics, dynamics, and mathematical modeling of the reactivity of the reactor system contributes immensely to the overall plant operations. Hence, the introduction of simulation and prediction of the system's behavior are taken into consideration. This helps to compare our results to existing data, design new processes, analyze and improve on the existing data. However, in some cases where a mathematical model is not available, it becomes difficult to use this method. Initially, when Artificial Intelligent Systems (AIS) based technology was not available, better machines and engineering designs were not developed. Today's world provides us with sensor technologies, hardware, and software systems to build intelligent systems. For instance, a highly interconnected network of a large number of processing elements called neurons in an architecture inspired by the human brain. The main aim is to transform inputs into meaningful output. This is termed the Neural – computing network or simply neural network (see Table 1, Table 2, Table 3, Table 4, Fig. .3, Fig. 11, Fig. 12, Fig. 13, Fig. 14, Fig. 15).

**Table 1:** Data used for the calculations.

Property	Value
Thermal power (P_th_)	3000 MW
Full Energy (E_f_)	200 MeV (3.2E-11)
Mass of fuel	25 ton/yr
Enrichment (x)	4.55%
Volume of core $V_{f}$	1.84.10^7^cm^3^
Density (UO_2_)	10.9 g/cm^3^
Fractional abundance of the density	4.5
$N_{A}$	6.023e+23

**Table 2:** Neutron cross section and half-lives for VVER Uranium Fuel nuclides.

Nuclide	$\sigma_{c}\left(barns\right)$	$\sigma_{f}\left(barns\right)$	$\sigma_{a}\left(barns\right)$	$T_{\frac{1}{2}}\left(y\right)$
U^235^	8.14	35.1100	43.25	7.04e+08
U^236^	6.4	0.2100	6.61	234e+07
U^238^	25.2	0.1000	27.61	4.47e+09
Np^237^	29.74	0.53	30.27	2.14e+06
Pu^239^	49.20	86.57	135.77	2.41e+06
Pu^240^	153.07	0.58	153.65	6.56e+03
Pu^241^	30.92	92.49	123.41	1.44e+01
Am^242^	130.43	564.78	677.21	1.41e+02
Cm^243^	49.03	0.45	49.48	7.37e+03

**Table 3:** Results from ANFIS.

	Concentration								
Time 10^6^ s	U235 10^20^	U236 10^20^	U237 10^20^	U238 10^20^	Pu239 10^20^	Pu240 10^20^	Pu241 10^20^	Am242 10^20^	Am243 10^20^
0	5.19	0	0	108.8	0	0	0	0	0
4.32	4.93	0.05	0.0004	105.4	3.03	0.17	0.03	0.00105	0.00016
8.64	4.67	0.10	0.0014	102	5.89	0.66	0.23	0.0162	0.0049
12.96	4.41	0.15	0.0032	98.53	8.49	1.43	0.75	0.0791	0.035
17.28	4.15	0.19	0.0056	95.1	10.93	2.45	1.71	0.241	0.144
21.6	3.88	0.24	0.0088	91.68	13.17	3.69	3.22	0.568	0.43
25.9	3.62	0.29	0.0126	88.27	15.2	5.11	5.35	1.13	1.008
30.24	3.36	0.34	0.0172	84.83	17.06	6.70	8.18	2.019	2.10

**Table 4:** Results from simulink.

	Concentration								
Time 10^6^ s	U235 10^20^	U236 10^20^	U237 10^20^	U238 10^20^	Pu239 10^20^	Pu240 10^20^	Pu241 10^20^	Am242 10^20^	Am243 10^20^
0	5.01	0	0	107.9	0	0	0	0	0
4.32	4.8	0.04	0.0003	104.4	2.98	0.13	0.025	0.001	0.00014
8.64	4.55	0.09	0.0013	101.08	5.68	0.63	0.21	0.014	0.0046
12.96	3.99	0.14	0.0029	98.11	8.82	1.4	0.72	0.076	0.033
17.28	3.89	0.19	0.0052	94.89	11.03	2.41	1.69	0.22	0.12
21.6	3.74	0.21	0.0082	91.12	13.78	3.65	3.18	0.53	0.4
25.9	3.5	0.26	0.01	87.92	15.8	5.1	5.3	1.10	1
30.24	3.05	0.31	0.013	84.49	17.95	6.67	8.14	2.00	2.08

To attain a better result in our quest to get a better design for reactor operations, a collection of these techniques is used in this paper to realize a better result of data planned to be used in our reactors [5]. Each technique has its distinct advantage when used in simulating data. The neural network has the integral advantage of being able to acclimate itself and its learning proficiencies. One good feature of Fuzzy Logic (FL) is the ability to take into account the existing uncertainty and impression of actual systems with the support of the fuzzy ‘*if-then’* rules. Focusing on these two techniques, there is a hybrid system that could be used to attain a more accurate result in our analyses. This is called the Adaptive neural-fuzzy inference system (ANFIS). The great expertise and knowledge exhibited in the fuzzy system display a better end preprocessor for the neural network input and output layers. From the literature, it is known that Neural Network (NN) learning procedures are used to regulate the parameters of the expert knowledge-based fuzzy system [6].

### 1.1 Background of simulink and ANFIS training data

#### 1.1.1 Simulink

This is a MATLAB –based graphical programming environment that is used for simulating, modeling, and analyzing systems that are dynamically formulated. The primary interface of this is the development blocks to represent the intended design. Simulink is used for many automatically controlled processing. One important aspect of Simulink is how it is used to model a non-linear system, which a transfer function cannot do. Simulink is integrated with MATLAB and hence there can be an easy transfer of data between the two programs [7].

#### 1.1.2 The adaptive neuro-fuzzy inference system (ANFIS) structure

**Fig. .1:**
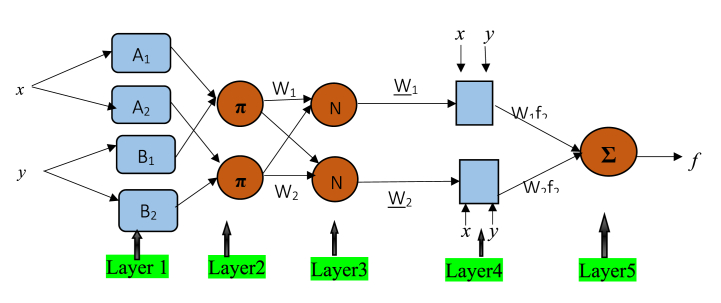
Type-3 ANFIS structure: Reasoning mechanism for Sugeno model.

ANFIS is based on a data procedure to represent a neural network method to solve problems. The procedure for the data simulation in ANFIS is inferred on a cluster in the training set of numerical samples [3,8]. For an illustration, see Fig. 1 Considering that the fuzzy system has two inputs and a single output, the rule, *‘if-then*’ of Sugeno in fuzzy is adopted. Take,

**Fig. .2:**
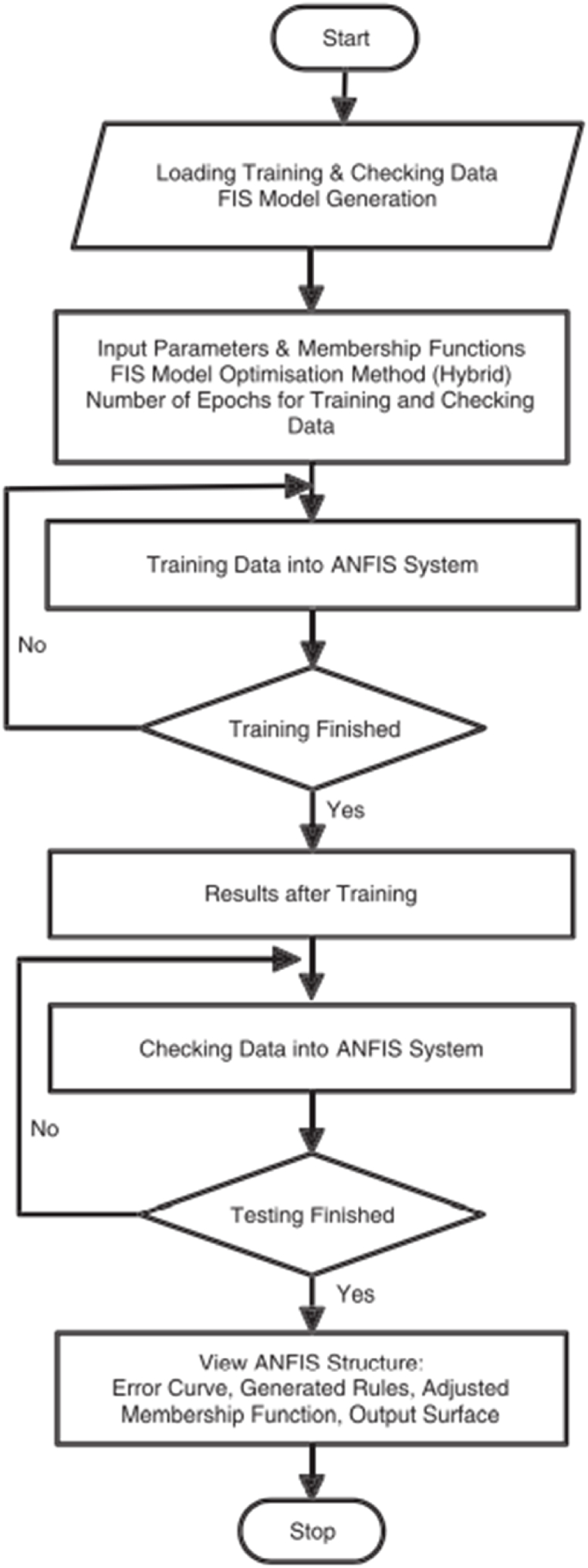
Method used in the ANFIS training system.

**Fig. .3:**
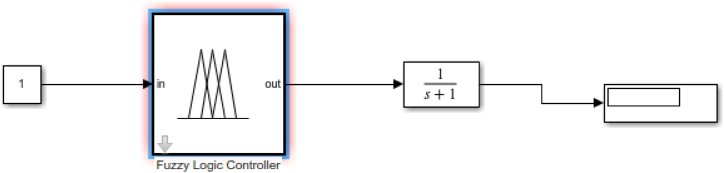
Tune-in the Sugeno controller.

Input 1 as *x*.

Input 2 as *y*.

Output as *f*, which is a crisp function of the inputs A and B.

Simply, if ‘*x*’ and ‘*y*’ are the fuzzy inputs in the antecedents, then the output *f* = f (*x, y*). The function f (*x,y*) is a polynomial function and approximates the out within the fuzzy region. A zero order in Sugeno fuzzy is modeled when f (*x, y*) becomes a constant. When the function f (*x, y*) is considered a first-order polynomial, a Sugeno fuzzy model of a first-order is formed as well. Here, two rules may be stated;

Rule 1: **if** *x* is A_1_ and *y* is B_1_ **then** *f*_*1*_ *= p*_*1*_*x + q*_*1*_*y + r*_*1*_

Rule 2: **if** *x* is A_2_ and *y* is B_2_ **then** *f*_*2*_ *= p*_*2*_*x + q*_*2*_*y + r*_*2*_

A Suge no-suggested type-3 inference system is used where each rule has an output which is a linear combination of the inputs. The weighted average w_1_ of each rule's output contributes to the final output. This is represented below.

Where.

• *A*_*1*_ and *B*_*1*_ are the set of fuzzy.
• *f*_*i*_ are the outputs that are found within the fuzzy area which are specified by the rules
• *p*_*i*_*, q*_*i,*_*and r*_*i*_ are the design parameters that were determined when the training was done

In ANFIS training, there is no way an output function will be shared to different rules the same there should be an equal number of rules to the number of memberships [6].

Interpretation of the layers.

**Layer 1:** The nodes seen in this layer are all adaptive nodes. The outputs of this layer are translated as the fuzzy membership grades of the inputs. It is mathematically expressed as

$$O_{1,i}=\mu_{Ai}\left(x\right),i=1,2$$

$$O_{1,i}=\mu_{\beta i-2}\left(y\right),i=3,4$$

The *A*_*i*_ and *B*_*i*_ values are the linguistic labels, (ie, high, average, low, etc.), and *x* and *y* are the inputs to node *i*. These can adopt to any fuzzy membership functions.

Layer 2: Layer 2 depicts fuzzy operators whose nodes are fixed. The ***and*** operator tool is used here to fuzzily the inputs. To show that they perform as a more straightforward multiplier, it is labeled with the **π** symbol. It calculates the firing strength, which is *w*_*i*_ of a given rule. In simple terms by multiplying all the incoming signals, the output value is found. The input is represented as;

$$O_{2,i}=w_{i}=\mu_{Ai}\left(x\right)*\mu_{Bi}\left(y\right),i=1,2$$

Layer 3: It is also a fixed node and it is represented by N. The *i*^*th*^ node will compute the ratio of the rule firing strength of the same *i*^*th*^ node. Here, its output values can be termed normalized firing strength. Mathematically given as,

$$O_{3,i}={\bar{w}}_{i}=\frac{w_{i}}{w_{1}+w_{2}},i=1,2$$

Layer 4: To obtain the output of the node, the normalized firing strength is multiplied by the first-order polynomial in the Sugeno model. The nodes are adaptive as well. It is given by,

$$O_{4,i}={\bar{w}}_{i}f_{i}={\bar{w}}_{i}\left(p_{i}x+q_{i}y+r_{i}\right),i=1,2$$

Layer 5: in layer 5, the summation symbol ∑ is used. This node sums all incoming signals to give out the output. This is termed the overall output. It is mathematically represented as,

$$O_{i}^{5}=overall\;output=\sum_{i}\bar{w_{i}}f_{i}=\frac{\Sigma_{i}w_{i}f_{i}}{\Sigma_{i}w_{i}}$$

#### 1.1.3 ANFIS training procedure

First of all, a training data should be obtained which should have inputs/output pairs. The number of inputs depends on what purpose one intends to use them for. This training data is a set of vectors. In ANFIS, two vectors are used in the training. As explained earlier, the overall output depicts the outcome of the results. The next values are found by applying the least square method after the limit between outputs for the actual values and the desired values. However, if the error found is larger than the verge value, the principal parameter will be reorganized using the gradient descent method. If the outcome of the error is less than the verge value, the process is said to be accurate and it is terminated [7] (see Fig. 2).

## 2 Methodology

A simulink code was created which was used to estimate the flux value at the startup of the reactor. This was considered for static and dynamic processes. The dynamic process advances to help to know how the concentration of the considered nuclides changes with time.

Simulink blocks were developed using Bateman's equation to estimate the concentration of the nuclides. The obtained values were trained in the ANFIS [2]. The ANFIS helped to tune in the values. This was accomplished using the Sugeno-block in the MATLAB simulink. This gave a more appropriate solution to the task as compared to the conventional values from mathematical calculations. The ANFIS trains and tests errors. After several pieces of training to estimate the RMSE, it reduced it to the minimum acceptable error level. The results are graphically shown by comparing both techniques used.

ANFIS gave the best performance for every training since it combined two training systems, which are, the neural network training system and the Fuzzy inference system. Suitable training data were initially created to enable possible data training in the Neuro-Fuzzy system. It used the ***anfis*** function for the training and the ***evalfis*** function to evaluate the system and eventually compared it to the desired output [9].

The data prepared to be used in training the *anfis* in MATLAB was in a matrix form. The last column became the output value and the other columns before that were considered the input(s). Depending on the training system, the number of input values can be many, however, the output value is always one. The command *genfis1* from MATLAB helped to create an initial set of membership functions. The system began the training immediately after the initial membership functions were created. The defined training data, then the *fismat* command were used to generate the membership functions. The training error and the final membership functions were produced once the training was complete. Checking data was loaded into the system to enhance the perfection of the outcomes, especially in reducing larger errors. Though only the training data for the ANFIS gave good results, but the checking data will enhance the system to understand the training better and perform perfectly [6,9].

The *evalfis* is enacted into action after the system training was complete. This was where system performance evaluation was done. Here, a set of input data were put into the system. The system automatically gave its output values. The values obtained were measured by the correlation between the learning content and the desired contexts.

Neutron flux and Nuclide concentration simulation (block diagram) in the reactor core: Developing the code.

The flux into the reactor is given using the equation [10,11].

(1) $$\varphi =\frac{P_{th}}{{(\Sigma }_{f}^{U5}+\Sigma_{f}^{U8})V_{f}E_{f}}$$

Where;

φ−neutronflux.

$P_{\text{th}}-\text{thermal}\;\text{power}$.

$\Sigma_{f}^{U5}-\text{macroscopic}\;\text{fission}\;\text{cross}\;\text{section}\;\text{uranium}\;235$.

$\Sigma_{f}^{U8}-\text{macroscopic}\;\text{fission}\;\text{cross}\;\text{section}\;\text{of}\;\text{Uranium}\;238$.

$V_{f}-\text{volume}\;\text{of}\;\text{the}\;\text{core}$.

$E_{f}-\text{full}\;\text{energy}\;\text{at}\;200\text{MeV}$.

$$\text{But}\;\text{generally};\;\Sigma_{f}^{U}=\sigma_{f}N_{o}$$

Where;

$\sigma_{f}=\text{microscopic}\;\text{fission}\;\text{cross}\;\text{section}\;\text{of}\;\text{the}\;\text{nuclide}$.

$N_{o}=\text{initial}\;\text{concentration}\;\text{of}\;\text{the}\;\text{nuclide}$.

$$\text{Also}\;N_{o}=\frac{\rho N_{A}}{\mu }$$

Where;

ρ=densityoftheuraniumfuel.

$N_{A}-\text{avogadros}\;\text{constant}$.

μ−molarmassofthenuclide(s).

Data used from library are as follows. For the microscopic fission cross-sections, one group of neutron cross-sections was considered. It could be trusted that using one group of neutron data, the fuel in the future could be changed to a MOX fuel and would not affect the final parameters [10].

The equations below are used for the uranium fuel cycle.

(2) dNU235dt=N0U235·(1−Φ·σaU235‾);

(3) NU236dt=N0U236+(N0U235·Φ·σcU235‾−N0U236·Φ·σaU236‾)

(4) NU237dt=N0U237+(N0U236·Φ·σcU236‾−N0U237·Φ·σaU237‾)·

(5) NU238dt=N0U238·(1−Φ·σaU238‾);

(6) NPu239dt=N0Pu239+(N0U238·Φ·σcU238‾−N0Pu239·Φ·σaPu239‾)

(7) NPu240dt=N0Pu240+(N0Pu239·Φ·σcPu239‾−N0Pu240·Φ·σaPu240‾)

(8) NPu241dt=N0Pu241+(N0Pu240·Φ·σcPu240‾−N0Pu241·Φ·σaPu241‾)

(9) NAm242dt=N0Am242+(N0Pu241·Φ·σcPu241‾−N0Am242·Φ·σaAm242‾)

(10) NAm243dt=N0Am243+(N0Am242·Φ·σcAm242‾−N0Am243·Φ·σaAm243‾)

## 3 Results and discussion

The average flux value was φ= 2.639 × 10^14^

**Fig. .4:**
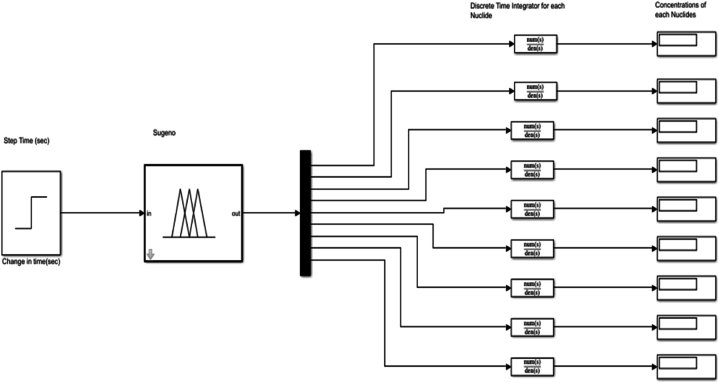
The ANFIS transient calculation of each nuclide concentration.

For this research, the atomic number density (atomic weight) and the neutron flux affected the power reactor. One affects the other in the reactor operation. Over a short time, the atomic density of the atoms in the fuel remains unchanged, which means the fission reaction has not taken much effect on the reactor operation [3]. This occurs when the reactor operates at a constant power level. However, for fissile isotopes, the atomic number densities decrease over a period of months. This happens due to fuel burnup [11]. This also results in the decrease of the macroscopic cross-sections of the fissile nuclides. When the macroscopic cross-sections decrease the cross-sectional area within which the neutrons travel also decreases. It gradually slows down the increase in the neutron flux, just to keep the desired power level of the reactor [[6], [12]] (see Fig. 4).

**Fig. 5:**
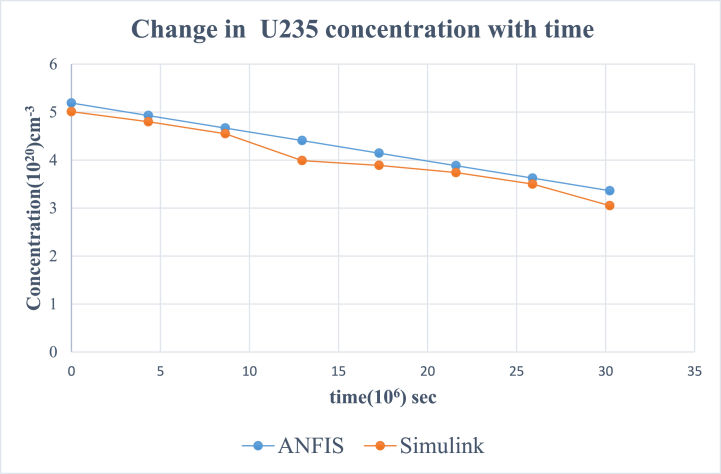
Change in U-235 concentration in uranium fuel.

According to the results, the two main nuclides considered at reactor start-up, U-235 and U-238, had their concentration values reducing with time. U-238(Fig. 8), depreciated more sharply than it was in U-235(Fig. 5). All the other nuclides had increments in their concentrations. Amongst them, Pu-239(Fig. 9) showed a significant increase over the rest of the nuclides. This is because Pu-239 becomes more fissionable at reactor operation. If U-235 increases more, it means the fuel will be more fissile and increase U-235 enrichment which will be more than the 3–5 % threshold for safe reactor operations [11,13]. The more U-235 gets concentrated, the more neutrons are released which may be captured by U-238 and eventually produce excess Pu-239, which is already increasing during fuel usage. Since the results gave a significant decrease in U-238 and U-235 and a moderate increase in U-239, the goal of the study was met (see Fig. 10, Fig. 11, Fig. 12, Fig. 13, Fig. 14, Fig. 15) [14].

**Fig. 6:**
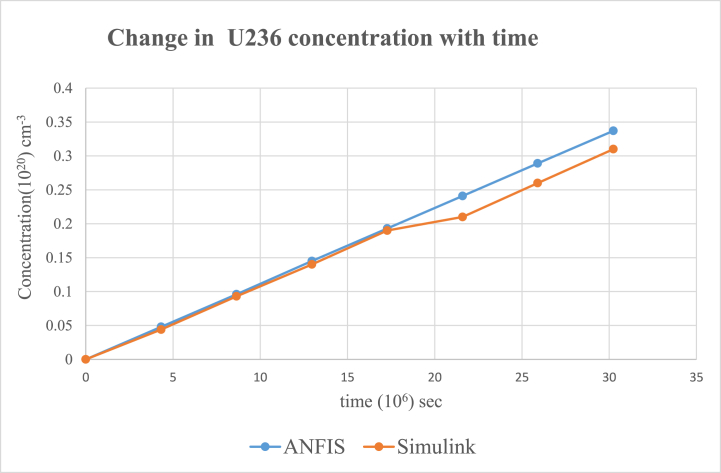
Change in U-236 concentration in uranium fuel.

**Fig. 7:**
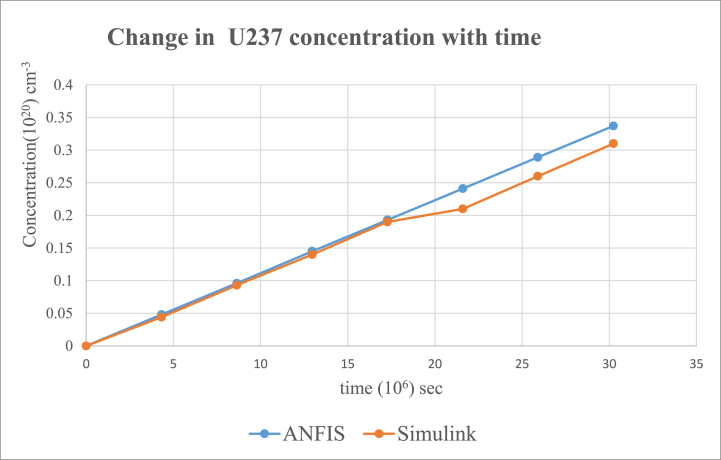
Change in U-237 concentration in uranium fuel.

**Fig. 8:**
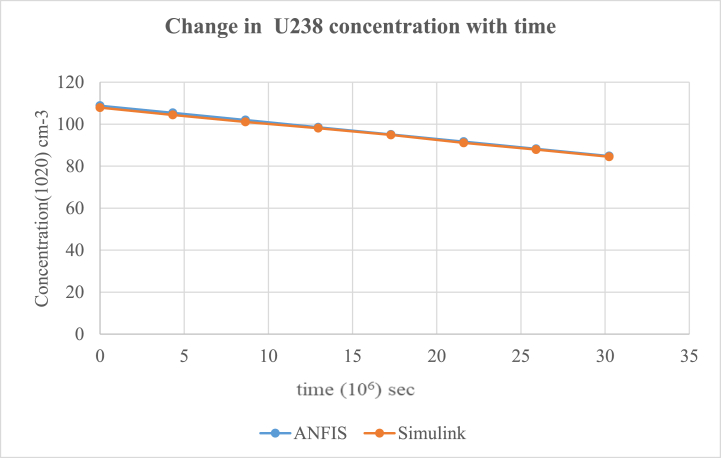
Change in U-238 concentration in uranium fuel.

**Fig. 9:**
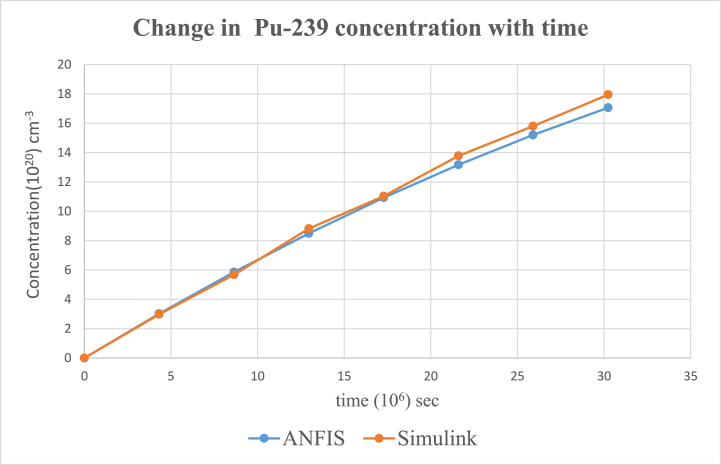
Change in Pu-239 concentration in uranium fuel.

**Fig. 10:**
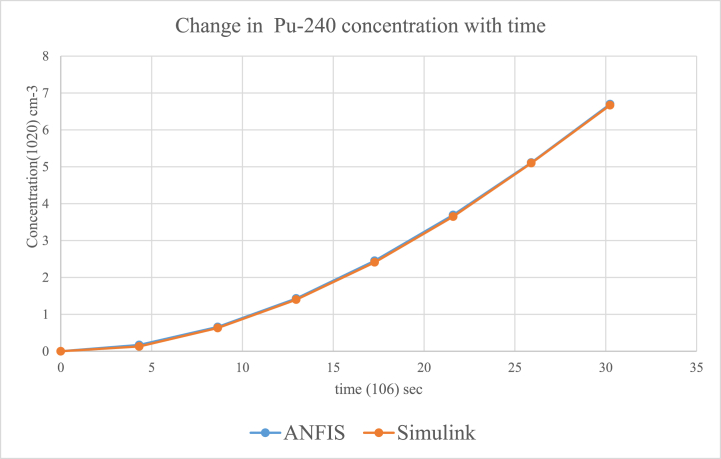
Change in Pu-240 concentration in uranium fuel.

**Fig. 11:**
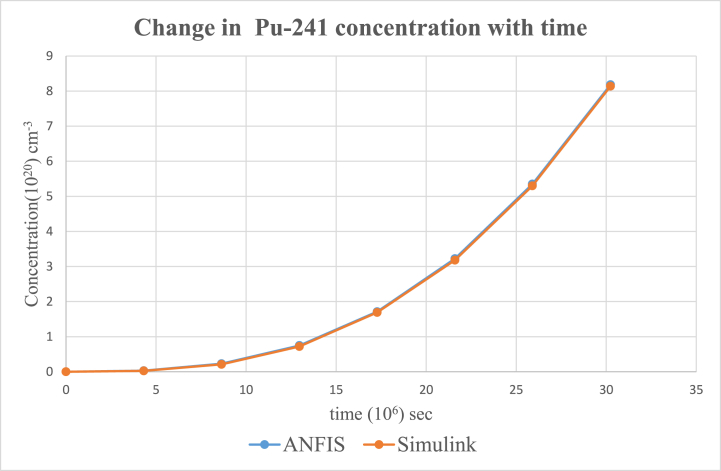
Change in Pu-241 concentration in uranium fuel.

**Fig. 12:**
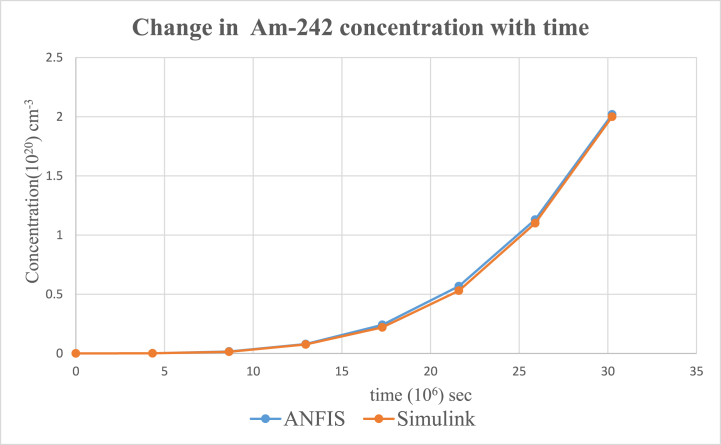
Change in Pu-242 concentration in uranium fuel.

**Fig. 13:**
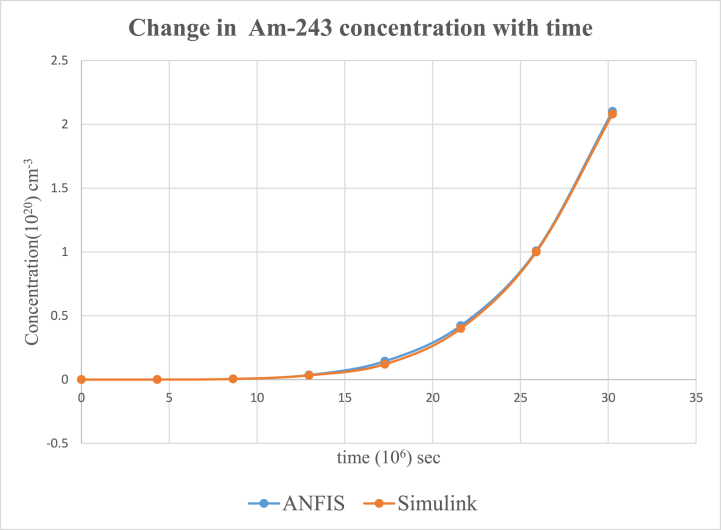
Change in Pu-243 concentration in uranium fuel.

**Fig. 14:**
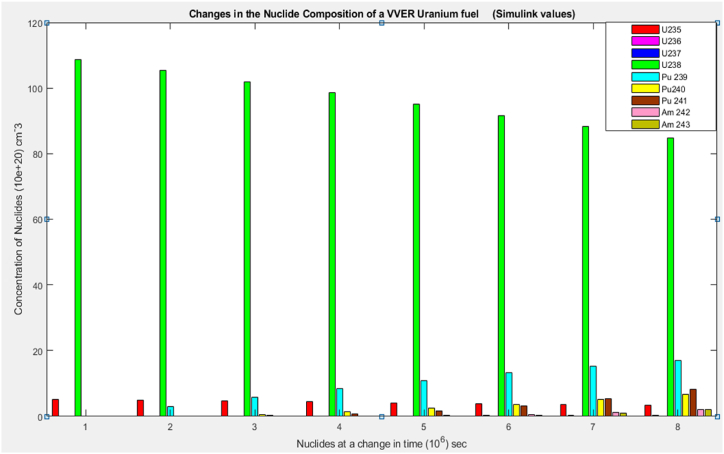
Change in the uranium fuel nuclides concentration (Simulink).

**Fig. 15:**
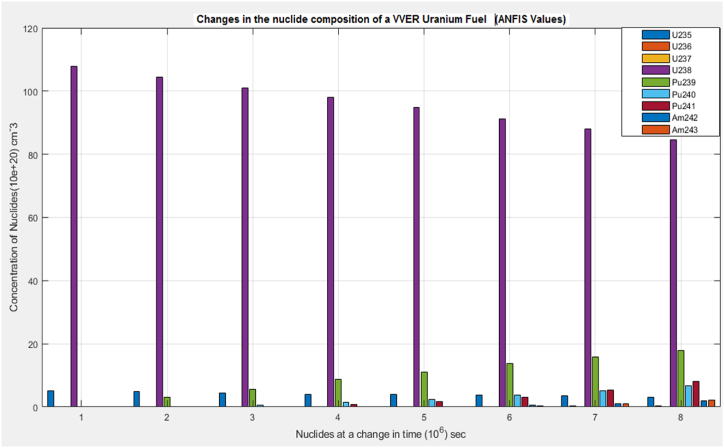
Change in the uranium fuel nuclides concentration (ANFIS).

### 3.1 Error analysis

The error analysis between the values obtained in MATLAB Simulink and the tuned-in values in the ANFIS after training and testing them has been made.

The root-mean-square error formula used is;

(11) $$RMSE={\left[\frac{\sum_{i=1}^{N}{\left[X_{i}\left(\mathrm{Exp}\right)-X_{i}\left(pred\right)\right]}^{2}}{N}\right]}^{0.5}$$

where,

N- the number of data.

$X_{i}\left(\text{sim}\right)$ – the Simulink values

$X_{i}(\text{pred}$)– the ANFIS results

In the analysis, it was observed that the differences in the values were acceptable and hence the trend of concentration change with time was in good agreement. An RMSE of about 0.98% and 1.25% for the ANFIS and simulink respectively [7]. Again the trends of concentration changes of both were in good agreement with the IAEA CAIN code [15].

## 4 Conclusion

Results from the simulink and ANFIS were compared. They both made a good agreement with a maximum error of about 0.98% and 1.25% for the ANFIS and simulink respectfully. However, there were a few inconsistencies with the analytical simulink values, which did not give straight lines as shown on the graphs for U-235(Fig. 5), U-236(Fig. 6), U 237 (Fig. 7) and U-239(Fig. 9). Again, the results were compared to the calculating Actinide Inventory (CAIN) code from the IAEA-TECHDOC-1535 published in 2007. This also showed a good agreement. The generated code in ANFIS for this research has an advantage. Which is, its ability to be run in the shortest time but still give accurate results. This is because ANFIS uses a multifaceted method in its calculations. The developed model will allow technologists to quickly perform calculations for the reactor, which is essential for safety systems. Hence ANFIS is the better option. The future is bright for further research into the use of Pu-239 + U-238 (MOX fuel) since Pu-239 was estimated to be the most generated nuclide. The fast-growing interest in the use of MOX fuel continuously in N.P.P. without a reactor shutdown has globally been accepted. Hence, very appropriate to research, developing a more effective and safest way of using MOX fuel alongside uranium fuel.

## Funding

There is none.

## Author contribution statement

All the authors listed have contributed significantly to the conception and design of the experiment, performed the experiment, analysed and interpreted the data, contributed reagents, materials, analysis and the writing of this article.

## Data availability statement

The data used is confidential.

## Additional information

No additional information is available for this paper.

## Declaration of competing interest

The authors declare that they have no known competing financial interests or personal relationships that could have appeared to influence the work reported in this paper.
